# Lectures on the Diagnosis and Treatment of Diseases of the Heart

**Published:** 1841-11-20

**Authors:** W. W. Gerhard


### MEDICAL EXAMINER.

DEVOTED TO MEDICINE, SURGERY, AND THE COLLATERAL SCIENCES.

No. 47.] PHILADELPHIA, SATURDAY, NOVEMBER 20, 1841. [Vol. IV.

### LECTURES ON THE DIAGNOSIS AND TREATMENT OF DISEASES OF THE HEART.

BY W. W. GERHARD, M. D.

### LECTURE XIX.

Not only the sounds of the heart, but the rhythm or succession offer points of interest for diagnosis. It is very clear that as the sounds are, in a normal state, separated by well marked divisions of time, a disorder of the rhythm or successsion can only arise from some material obstruction to the action of the heart, and the play of its valves, or from some decided functional disorder. The latter can produce only a moderate disturbance; such as irregularity in the relative rapidity of the pulsation, with occasional interruption of a single beat; or, at most, the heart may pulsate in an intermittent manner, a pulsation being from time to time absent, at intervals which recur with some regularity. The distinction between an irregular and an intermittent action of the heart is mainly the recurrence as to time of the latter symptom, and the variableness of the former. An intermittent pulsation of the heart is congenital or nearly so with many individuals, lasting through a long life without much disorder of the general health; but if we watch these individuals narrowly, we shall find that most of them at last suffer in some way from organic diseases of the heart. The temporary irregularity of the pulsation is much less important. Under many circumstances it is rather a favourable symptom, and occurs frequently at the termination of acute diseases, especially of those which have a definite duration, such as the exanthemata. There are other cases in which the irregularity is really a pathological symptom, but refers to another organ than the heart. This is the case with inflammations, or other diseases of the brain, which in many stages of their progress are attended with irregularity of the pulse. There is, however, another set of cases in which the irregular action of the heart is a sign of disease of the organ itself, and if it be connected with other and more decided indications of inflammation, or more permanent organic alterations, it has its value. But as in itself, irregularity is insignificant, the importance of the symptom in the study of heart disease is extremely slight.

There is another alteration of the rhythm very different from those just alluded to, and of much graver moment. In fact, it is almost confined to organic valvular disease, and mainly to concretions at the mitral valve: the proportion, as well as the peculiar character o the sounds, is then nearly destroyed, and we have a confused churning or purring sound, without any distinction of the first or second. So complete a destruction of the ordinary sounds of a healthy heart indicates the gravest lesions, and is generally connected both with dilatation of the cavities and disease of the valves.

The purring sensation (*fremissement cataire*) often felt as well as heard at the region of the heart, belongs almost as appropriately to this part of the subject as to any other. It is a sign of gravity, because the total change in both impulsion and sound which accompanies it can scarcely occur without both valvular and muscular disorder, and a free passage is opened for the blood, which is thus broken into many currents. The regular action of the heart is broken up, because there is no longer a uniform point of resistance, nor of repose; for the stream of blood is no longer cut off by the valves. The purring is quite characteristic of the sign, both as descriptive of the sensation of touch and of sound.

Another important sign of the diseases of the heart is derived from the mode of its contraction. Instead of the natural contraction, we may find it to be quick, jerking, and spasmodic, or it may be confused and indistinct. These are peculiarities very difficult to describe, and only to be appreciated by one who has been long practiced in the observation of the healthy heart. After a knowledge of the natural contraction is acquired, any deviation from it becomes very apparent. When the internal membrane of the heart is inflamed, the contractions lose their sharpness and distinctness, and succeed each other in a confused, jerking manner: the sign is much the same in cases in which the valves are much diseased, especially when there is great dilatation of the auriculo-ventricular openings. Indeed any decided organic lesion of the heart modifies the natural action, and even functional disturbance of it to a certain extent produces the same effect, but in a less degree, and for a less period of time.

The next part of our subject leads us naturally to the study of the individual diseases of the heart.

